# Prechoroidal cleft thickness correlates with disease activity in neovascular age-related macular degeneration

**DOI:** 10.1007/s00417-021-05384-w

**Published:** 2021-09-07

**Authors:** Mariano Cozzi, Davide Monteduro, Salvatore Parrulli, Federica Ristoldo, Federico Corvi, Federico Zicarelli, Giovanni Staurenghi, Alessandro Invernizzi

**Affiliations:** 1 Eye Clinic, Department of Biomedical and Clinical Science “Luigi Sacco”, Luigi Sacco Hospital, University of Milan, Via G.B. Grassi, 74 - 20157 Milan, Italy; 2 Faculty of Health and Medicine, Save Sight Institute, University of Sydney, Sydney, NSW Australia

**Keywords:** Prechoroidal cleft, Pigment epithelial detachment, Neovascular age-related macular degeneration, Macular neovascularization, Optical coherence tomography, Exudation

## Abstract

**Purpose:**

The purpose of this study was to investigate the structural variations of the hyporeflective pocket of fluid (prechoroidal cleft) located between Bruch’s membrane and the hyperreflective material within the pigment epithelial detachment (PED) in patients with neovascular age-related macular degeneration (nAMD).

**Methods:**

In this retrospective, observational case series study, patients diagnosed with nAMD and prechoroidal cleft associated with other activity signs of the macular neovascularization (MNV) were included. Structural optical coherence tomography (OCT) scans were evaluated to obtain anatomical measurements of prechoroidal cleft and PED at three different visits (T0, inactive MNV; T1, active MNV; T2, treated inactive MNV). The variations in size of the cleft and the PED were correlated with nAMD activity.

**Results:**

Twenty-nine eyes from 27 patients were included. The subfoveal measurements showed a significant increase of prechoroidal cleft height and width from T0 to T1 (*P* < 0.05) and a subsequent decrease of the cleft height after treatment with anti-VEGF agents (*P* = 0.004). A similar significant trend was observed for the greatest prechoroidal cleft height and width, obtained assessing the whole OCT raster. In the multivariate analysis, the cleft height was significantly affected by both time (*P* = 0.001) and PED height (*P* < 0.0001). By contrast, the effect of fibrovascular tissue size within the PED was not significant. Visual acuity did not correlate with prechoroidal cleft size.

**Conclusion:**

Prechoroidal cleft increased in association with MNV reactivation and decreased after treatment. Our results suggest that prechoroidal cleft could represent an accumulation of fluid actively exudating from the MNV and should be considered a sign of nAMD activity.

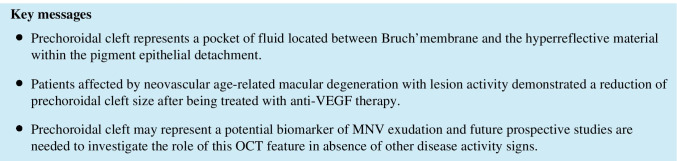

**Supplementary Information:**

The online version contains supplementary material available at 10.1007/s00417-021-05384-w.

## Introduction

Neovascular age-related macular degeneration (nAMD) is currently monitored using optical coherence tomography (OCT) and the presence of certain signs on OCT scans is associated with active macular neovascularization (MNV) [1]. In particular, the identification of intraretinal and/or subretinal fluid and/or subretinal hyperreflective material (SHRM) is widely considered a sign of disease activity and guides treatment decisions such as anti-vascular endothelial growth factor (VEGF) injections administration in patients with a pro-re-nata regimen (PRN) [2] or treatment interval shortening in treat and extend schemes [3].

Some OCT signs like the presence of a retinal pigment epithelial (RPE) detachment (PED) can be associated with the presence of an MNV in most cases, but their identification does not always correlate with disease activity [4, 5]. In 2014, a new OCT finding characterized by the presence of hyporeflective space between Bruch’s membrane (BM) and hyperreflective materials within PEDs in patients affected by MNVs has been described for the first time [6]. This particular space initially simply named “cleft”[7, 8] has been further characterized in chronic fibrovascular PED[6, 9] and defined “prechoroidal cleft” [9] despite been located above the BM.

Prechoroidal clefts are reported to affect up to 8.1–22.3% of the eyes under treatment for neovascular AMD [10]. In particular, this OCT feature is only present within a PED. Independent risk factors have been associated with their development, with a higher incidence in eyes with type 3 MNV and typical AMD being more prone to show this optically empty space compared with polypoidal choroidal vasculopathy [11]. Early development of the cleft has also been associated with worse vision in the long-term [12]. The origin of the cleft has been attributed to a possible accumulation of the fluid generated by the fibrovascular tissue [6]. However, its correlation with the lesion activity and treatment remains unknown.

The present study aimed to investigate the structural response of prechoroidal cleft to anti-VEGF treatment in patients affected by nAMD and to find the possible correlation between the cleft extent and the activity of the MNV.

## Methods

This was a retrospective, single-center, observational study. Clinical charts and imaging studies of patients with nAMD followed at the Eye clinic, Department of Biomedical and Clinical Science “L. Sacco,” Luigi Sacco Hospital, University of Milan, Milan, Italy under treatment between 2018 and 2019 were reviewed. To be included in the study the eyes had to:

1. be diagnosed with nAMD.
2. have an inactive MNV defined as a MNV that had received no treatment for at least 3 months and still had no signs of activity. The 3-month “wash-out” was established on the basis of the pharmacokinetics of anti-VEGF agents in order to minimize drugs' effect on the neovascular complex anatomy [13].
3. have a MNV activation with a visible prechoroidal cleft on OCT detected at the first visit performed after point (2).
4. be treated with anti-VEGF agents according to a PRN regimen.
5. have follow-ups performed on the same OCT device (Heidelberg Spectralis, Heidelberg Engineering, Heidelberg, Germany) with the tracking system set up to repeat the scans in the exact same location at different visits;

Exclusion criteria were considered the presence of massive subretinal hemorrhages, RPE tears, MNV treatments other than anti-VEGF therapy (e.g., photodynamic therapy) before and during the study period considered in the analysis, and inadequate scan quality.

We included three different visits (T) in the analysis of the prechoroidal cleft: (T0) non-active MNV with a washout of anti-VEGF agents as described above, (T1) considered the following OCT scan performed after T0 with evidence of MNV activity and presence of prechoroidal cleft, (T2) considered the first visit after T1 with no signs of MNV activity following anti-VEGF treatment.

The study was approved by the local institutional ethics committee and was conducted in accordance with the tenents of the Declaration of Helsinki. Signed consent was obtained from each participant.

At each visit, all patients underwent a complete ophthalmic examination including best correct visual acuity, slit-lamp examination, fundus biomicroscopy, and spectral domain OCT.

The OCT acquisition protocol included 49 horizontal B-scans, each composed of at least 9 averaged frames covering a 20 × 20° area. Prechoroidal cleft was defined as the presence of hyporeflective space located between hyperreflective material adherent to the basal surface of the elevated RPE (fibrovascular tissue) and the underlying BM [6].

Medical records were analyzed collecting demographic data, best-corrected visual acuity reported as Early Treatment Diabetic Retinopathy Study (ETDRS) letter score, number and type of intravitreal injections administered. Other data were also collected based on the images performed on the baseline visit. In particular, we evaluated the following: neovascularization subtype, presence of either drusen or subretinal drusenoid deposits, presence of complete RPE and outer retinal atrophy (cRORA) [14], fibrosis [15], and prechoroidal cleft.

**Fig. 1:**
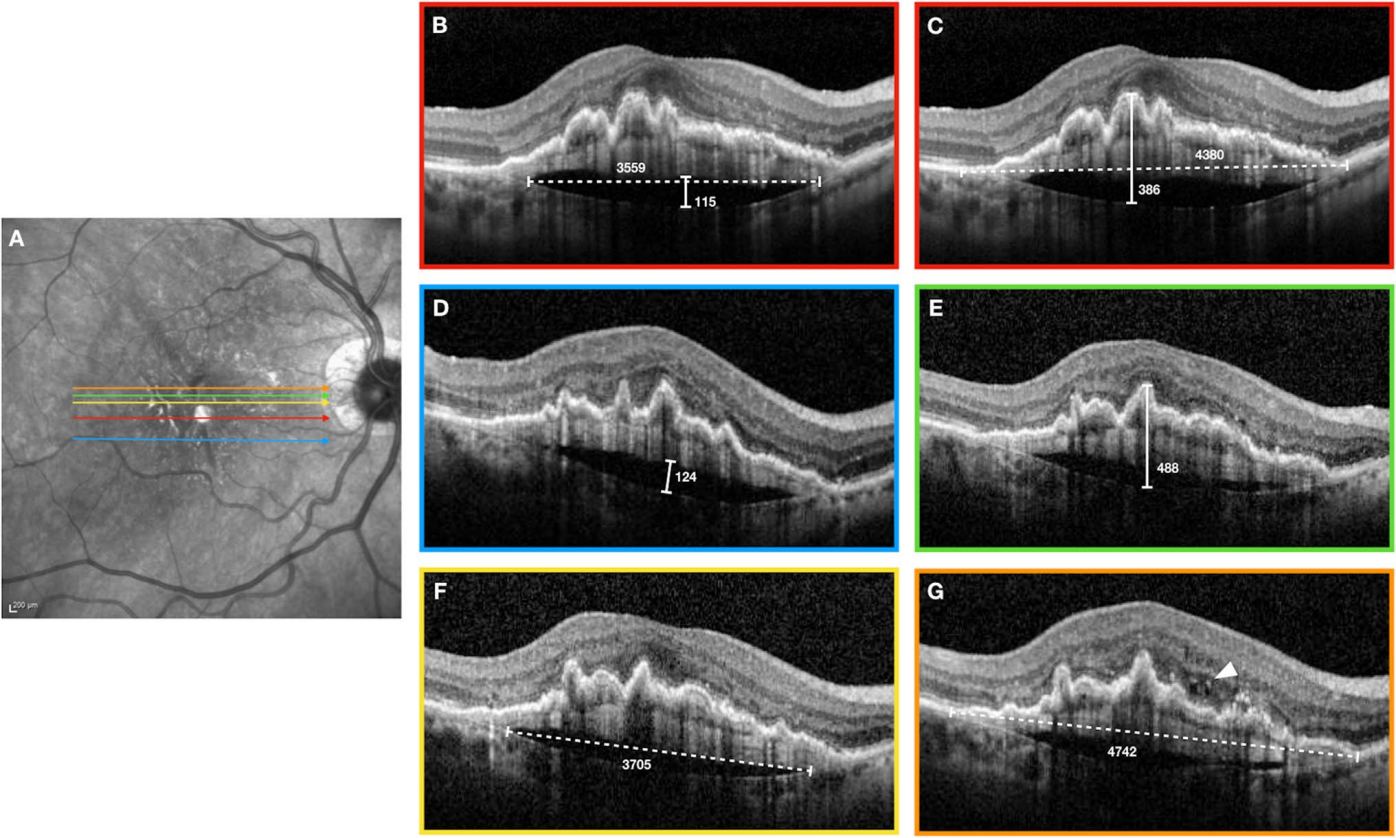
Reference boundaries in a study patient showing measurements obtained in T1 (active MNV). **A**—Near-infrared reflectance of the fundus with superimposed colored lines which correspond to the position of the structural optical coherence tomography (OCT) B-scans. **B**—Measurements of subfoveal prechoroidal cleft maximum height (solid line) and subfoveal prechoroidal cleft width (dotted line). **C**—Measurements of subfoveal pigment epithelial detachment (PED) maximum height (solid line) and subfoveal PED width (dotted line). **D**—Measurements of greatest prechoroidal cleft height. **E**—Measurements of the greatest PED height. **F**—Measurements of the greatest prechoroidal cleft linear dimension. **G**—Measurements of the greatest PED linear dimension. This last B-scan also shows evidence of intraretinal cysts (arrow head) representing MNV activity. All measurements are obtained using the in-built measure distance tool (Eye Explorer version 1.9.10 (Heidelberg Engineering, Heidelberg, Germany)) and are expressed in microns

At each of the three visits considered in the study (T0, T1, T2), we measured the following features on OCT: subfoveal prechoroidal cleft maximum height, subfoveal prechoroidal cleft width, the greatest prechoroidal cleft height, and the greatest prechoroidal cleft linear dimension. Moreover, to study the prechoroidal cleft in the context of the PED, we measured the following: subfoveal PED maximum height, subfoveal PED width, the greatest PED height, and the greatest PED linear dimension (horizontal). The exact reference boundaries considered to perform these measurements are presented in Fig. 1.

Subfoveal measurements were obtained from the B-scan passing through the center of the fovea using the same reference for all the visits (T0-T1-T2) and consisted of the greatest linear dimensions for both the horizontal and vertical plane of the B-scan. The other four measurements had to describe the greatest extent of the cleft or the PED whose location could vary with time. Thus, these measurements were not necessarily performed on the same scan at different time points. A similar methodology was previously adopted in studies with a similar design [16, 17].

The height of the fibrovascular tissue within the PED was calculated by subtracting the cleft height from the PED height. This value was only obtained on the foveal scan and was used in the analysis to assess the possible effect of the fibrovascular component extent on the cleft extent.

All measurements were performed by two independent graders (SP and DM) using the in-built measure distance tool (Eye Explorer version 1.9.10 (Heidelberg Engineering, Heidelberg, Germany)). Interobserver agreement was calculated.

### Statistical analyses

Normal distribution of the data was tested for all quantitative variables before the analysis. Results are reported as mean and standard deviation (SD) for continuous variables while categorical variables are presented as number and percentage.

Pairwise comparison was performed with Bonferroni correction to analyze the change of anatomical features (subfoveal prechoroidal cleft maximum height, subfoveal prechoroidal cleft width, subfoveal PED maximum height, subfoveal PED width, greatest prechoroidal cleft height, greatest prechoroidal cleft linear dimension, greatest PED height, and greatest PED linear dimension) and BCVA at different time points.

A multivariate regression analysis was used to investigate the effect of the time (T0, T1, T2) and PED on the height of the prechoroidal cleft and the effect of the time (T0, T1, T2) and fibrovascular tissue on the height of prechoroidal cleft.

The intraclass correlation coefficient (ICC) was calculated in order to assess interobserver variation in calculating the anatomical variables.

Statistical analysis was performed using R statistical package version 3.3.1 (R Project–The R Foundation for Statistical Computing; http://www.R-project.org). A *P* value < 0.05 was considered to be statistically significant.

## Results

### Study patients

A total of 29 eyes from 27 patients (10 males and 17 females; mean age 77.5 ± 8.0 years) fulfilled the inclusion criteria were included in this retrospective analysis. Intraclass correlation coefficient showed excellent agreement between the two readers for all measurements, ranging from 0.81 (95% CI, 0.78–0.86) to 0.93 (95% CI, 0.87–0.96) (Supplementary Table). For this reason, measurements from Grader #1 (SP) were used for further analysis.

### Clinical characteristics at the diagnosis

Among the included eyes, 25 had a type 1 MNV (86.2%), one eye had a mixed type 1 and type 2 MNV (3.4%), and three eyes had a type 3 MNV (10.4%). At this stage, prechoroidal cleft was present in 11 eyes (37.9%). Other morphologic characteristics observed before starting the treatment are reported in Table 1.

**Table 1:** Demographic data and baseline clinical characteristics of patients enrolled in the study

Demographic and Baseline Clinical Characteristics		
Number of eyes, (patients)		29, (27)
Age (years) mean, (± SD), [range]		77.5, (±8.0), [64–91]
Gender	Males, *n* (%)	10, (37%)
	Females, *n* (%)	17, (63%)
MNV type	Type 1, *n* (%)	25, (86.2%)
	Mixed type 1 and 2, *n* (%)	1, (3.4%)
	Type 3, *n* (%)	3, (10.4%)
Baseline features	Drusen, *n* (%)	29, (100%)
	SDDs, *n* (%)	10, (34.5%)
	cRORA, *n* (%)	4, (13.8%)
	Fibrosis, *n* (%)	1, (3.4%)
	Prechoroidal cleft, *n* (%)	11, (37.9%)

*SD* standard deviation, *MNV* macular neovascularization, *SDDs* subretinal drusenoid deposits, *cRORA* complete retinal pigment epithelium and outer retinal atrophy

### T1 (active MNV) characteristics

At active (T1), the MNV showed the following features: 15 eyes (51.7%) had intraretinal fluid, 23 eyes (79.3%) had subretinal fluid, 12 eyes (41.4%) had SHRM, and 1 eye (3.4%) had an intraretinal hemorrhage. None of these features was detectable at T0 and T2 as the lesions could not show any sign of activity as per study protocol. At T1, all eyes showed the presence of a prechoroidal cleft as per inclusion criteria.

### Prechoroidal cleft

The subfoveal measurements showed a significant increase of prechoroidal cleft maximum height from T0 to T1 (*P* < 0.0001) and a subsequent decrease after receiving treatment (*P* = 0.0004). The same trend was observed measuring subfoveal prechoroidal cleft width from T0 to T1 (*P* = 0.048). However, no significant reduction was noticed from T1 to T2 (*P* = 0.164).

**Fig. 2:**
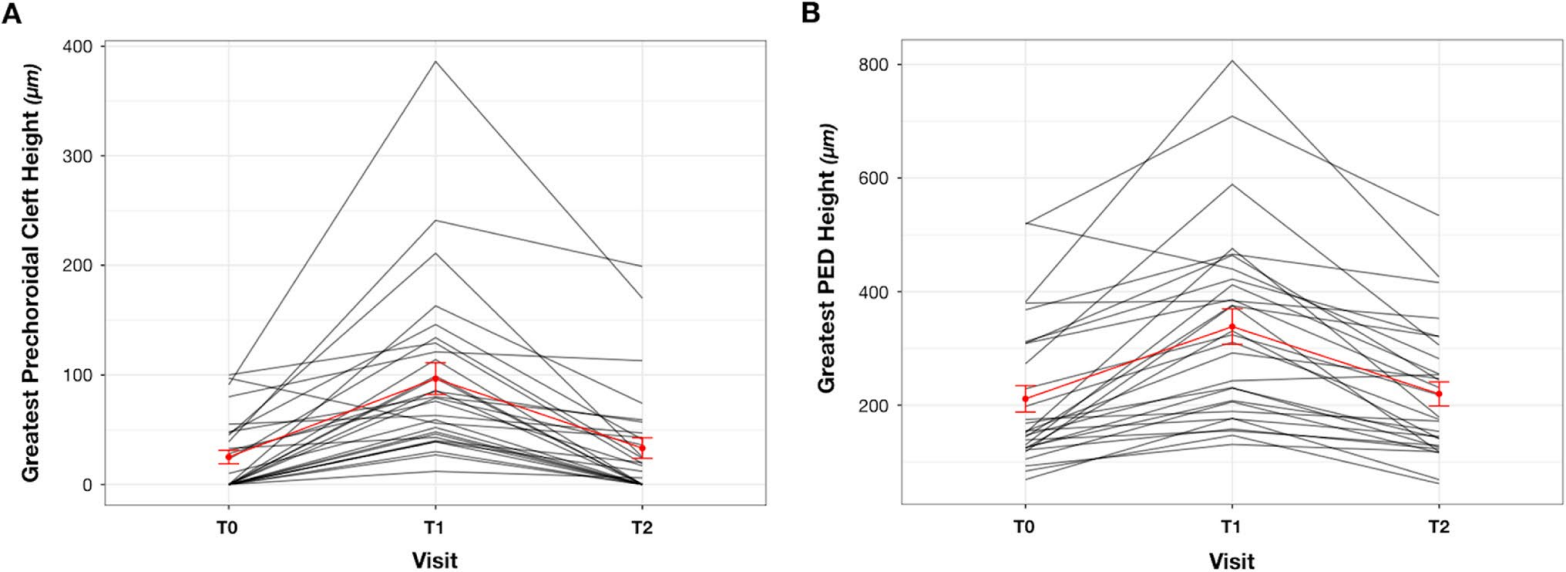
**A**—Graphical representation of greatest prechoroidal cleft height changes during the study period. **B**—Graphical representation of greatest pigment epithelial detachment (PED) height changes during the study period. Both of the diagrams show an increase of the anatomical feature during the disease activity and subsequent reduction in the inactive stage of the lesion

The greatest prechoroidal cleft height increased significantly from T0 to T1 (*P* < 0.0001) and decreased after treatment at T2 (*P* < 0.0001) (Fig. 2A). Similar to the subfoveal measurements, the greatest prechoroidal cleft linear dimension showed a significant increase from T0 to T1 followed by a similar decline from T1 to T2 (*P* = 0.0003). All the anatomical results are summarized in Table 2. In the multivariate analysis, the subfoveal prechoroidal cleft maximum height was significantly affected by both time (*P* = 0.0001) and subfoveal PED maximum height (*P* < 0.0001). By contrast, fibrovascular tissue height within the PED did not influence the subfoveal prechoroidal cleft maximum height significantly (*P* = 0.149).

**Table 2:** Spectral domain optical coherence tomography (SD-OCT) measurements of anatomical features at different MNV stages

SD-OCT features	T0	T1	T2	*P* value (T0–T1)	*P* value (T1–T2)
Subfoveal prechoroidal cleft maximum height (microns), mean (± SD)	20.38 (± 29.51)	61.55 (± 59.41)	27.52 (± 49.30)	** < 0.0001**	**0.0004**
Subfoveal prechoroidal cleft width (microns), mean (± SD)	600.10 (± 887.33)	1061.72 (± 969.41)	697.24 (± 1021.13)	**0.048**	0.164
Greatest prechoroidal cleft height (microns), mean (± SD)	25.07 (± 32.96)	96.59 (± 77.78)	33.17(± 50.37)	** < 0.0001**	** < 0.0001**
Greatest prechoroidal cleft linear dimension (microns), mean (± SD)	764.97 (± 1014.84)	1657.69 (± 885.20)	944.31 (± 1094.27)	** < 0.0001**	**0.0003**
Subfoveal PED maximum height (microns), mean (± SD)	170.55 (± 104.96)	279.45 (± 151.29)	181.10 (± 107.37)	** < 0.0001**	** < 0.0001**
Subfoveal PED width (microns), mean (± SD)	2894.83 (± 1494.24)	3020.03 (± 1373.34)	2970.41 (± 1491.11)	0.952	1
Greatest PED height (microns), mean (± SD)	211.31 (± 125.50)	338.45 (± 168.10)	219.90 (± 114.46)	** < 0.0001**	** < 0.0001**
Greatest PED linear dimension (microns), mean (± SD)	3193.10 (± 1273.39)	3493.03 (± 1009.93)	3426.45 (± 1200.42)	**0.007**	1

*SD-OCT* spectral domain optical coherence tomography, *SD* standard deviation, *PED* pigment epithelial detachment

Statistically significant *P* value is reported in bold

### PED and fibrovascular tissue within the PED

We found a significant increase between T0 and T1 for both subfoveal PED maximum height and greatest PED height (*P* < 0.0001). Both the parameters decreased significantly from T1 to T2 (*P* < 0.0001) (Fig. 2B). Conversely, the PED lateral extent did not show the same “increase–decrease” trend consistent with diseases activity across the three visits as it increased significantly in its greatest linear dimension from T0 to T1 (*P* = 0.007) but did not decrease from T1 to T2. All the anatomical results are summarized in Table 2.

The fibrovascular tissue within the PED significantly increased from T0 to T1 (*P* < 0.0001) and shrank after treatment from T1 to T2 (*P* < 0.0001) but these variations did not influence the prechoroidal cleft height significantly.

### Visual acuity

At T0, BCVA ranged from 30 to 85 letters, with a mean of 70.0 ± 13.7 letters (Snellen equivalent 20/40). At T1, the mean BCVA was 68.3 ± 14.2 letters (Snellen equivalent 20/40) while at T2, the mean BCVA returned to 70.0 ± 14.0 letters (Snellen equivalent 20/40). Comparing the BCVA among the three different visits, we found a significant decrease of 1.72 letters of the BCVA score (*P* = 0.007) between T0 and T1 and a significant increase of 1.72 letters (*P* = 0.007) between T1 and T2. By contrast, no significant difference was noticed between T0 and T2 (*P* > 0.05). Visual acuity did not correlate with the prechoroidal cleft size (*P* > 0.05).

## Discussion

In this study, we investigated the features of prechoroidal cleft in the context of nAMD and their possible correlations with disease activity. We observed that the prechoroidal cleft significantly increased in size (height and width) when the MNV showed signs of exudation and significantly reduced after treatment with intravitreal injections of anti-VEGF. Furthermore, the size of the cleft did not correlate with the size of the overlying fibrovascular tissue.

Prechoroidal cleft has been recently defined as a unique OCT feature in nAMD and nowadays there are conflicting theories about its clinical relevance [10]. Previous studies suggested a possible correlation between the cleft and MNV activity [6, 9], while others interpreted the cleft as a possible chronic structural alteration accompanying the neovascular tissue [9, 12], yet no studies investigated the variation of prechoroidal cleft size in association to nAMD activity and in response to anti-VEGF treatment.

**Fig. 3:**
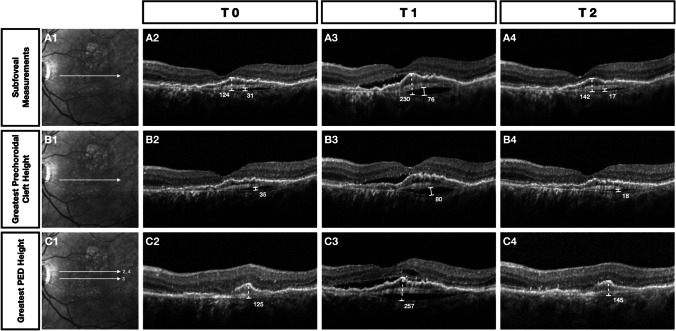
Clinical course of prechoroidal cleft and pigment epithelial detachment (PED) measurements in a patient enrolled in the study. A—Near-infrared reflectance with the corresponding position of the B-scans used in the analysis. A2–A4—Subfoveal PED maximum height (dotted line) and prechoroidal cleft maximum height (solid line) measurements at T0, T1, and T2. B2–B4—The greatest prechoroidal cleft height measurements at T0, T1, and T2. C2–C4—The greatest PED height measurements at T0, T1, and T2. The figure clearly demonstrates the presence of activity signs of macular neovascularization (MNV) at T1 corresponding to an increasing of either prechoroidal cleft or PED dimensions. After receiving one anti-VEGF injection, the last column illustrates the absence of activity signs with a considerable reduction of either prechoroidal cleft or PED dimensions. All measurements are obtained using the in-built measure distance tool (Eye Explorer version 1.9.10 (Heidelberg Engineering, Heidelberg, Germany)) and are expressed in microns

In our population, the size of the cleft increased significantly at T1, when the MNV was active and we obtained a significant decrease of both prechoroidal cleft height and width in the entire macular volume after treatment. These data suggest that the cleft could represent a pocket of fluid collected between the fibrovascular tissue and the Bruch’s membrane (BM). Similar to subretinal or intraretinal fluid, the fluid forming the prechoroidal cleft could have a variable response to the anti-VEGF treatment but its fluctuations seem related to the MNV exudation thus possibly serving as an adjunctive sign of disease activity(Fig. 3).

Pigment epithelial detachment is an established feature of nAMD [18, 19]. However, none of the latest multicentre trials has considered the PED size fluctuation as a sign of lesion activity. [20, 21] Furthermore, several studies demonstrated that PED is less responsive to anti-VEGF treatment compared to other fluid components. [18, 22, 23] This is likely related to the double component (i.e., fibrovascular and fluid) of the PED. In our population, we found a significant decrease of the PED height after receiving treatment with anti-VEGF agents while the lateral extension of the PED did not seem to be affected by the treatment. Furthermore, we observed that the variation in the PED height was significantly correlated with the variation in the prechoroidal cleft size while no associations were found with the fibrovascular component size. This suggests that the cleft could in fact represent an element of the PED better correlating with the MNV exudation and representing a superior sign of disease activity compared to the PED as a whole.

The presence of fluid detected by structural OCT is nowadays considered the key biomarker used for assessing lesion activity [24, 25]. The fluid may be identified at different levels such as intraretinal, subretinal, and sub-RPE. Post hoc analyses of clinical trials and real-world outcomes demonstrated that intraretinal fluid presence at baseline is associated with a worse visual prognosis [26, 27]. Subretinal fluid at baseline does not affect the outcome but its fluctuation during the treatment course is associated with a poorer prognosis [27–29]. Differently from these two components, fluid located underneath the RPE has not been directly associated with visual outcomes although once again, the eyes with higher fluctuations have shown a trend towards a poorer vision [30]. Considering the location of the cleft, the photoreceptors on top of it should not be affected by its presence [9, 11], but its fluctuations could still be relevant to management and treatment decisions.

A worse prognosis was described in a retrospective comparative study of eyes with an early developed cleft [12]. Overall, the mean VA of our cohort at T0 was similar to that at baseline (mean 70.0 ± 13.7 letters). We only considered the VA at the three-time points that were included in the analysis. Among these, we found a significant decrease of VA associated with lesion reactivation as expected, followed by an increase in VA after treatment. Previous studies reported prechoroidal clefts to be associated with a higher risk of RPE tear and subretinal hemorrhage [4, 6, 8, 11], but we did not observe such complication in our population during the study period.

In our population with prechoroidal cleft, most eyes were affected by type 1 MNVs (86.2%). This result partially reflects the distribution of MNV subtypes reported by Kim and coworkers in 2017 [12]. Type 3 MNVs have also been described as a risk factor for the development of prechoroidal cleft [11, 12]. However, only 10.4% of our clefts were associated with type 3 MNV. This disagreement could be explained by the different inclusion criteria between the studies. In fact, we included only patients diagnosed with prechoroidal cleft associated with other MNV activity signs.

Our study has some limitations that we would like to acknowledge. First of all, patients were included at different times during their treatment course and the time between the timepoints may vary according to the response to treatment. Secondly, the inclusion criteria were extremely rigid, and therefore, the eyes included in the study do not reflect the majority of nAMD with PED and our results should be considered with caution. On the other hand, inclusion criteria were designed in order to comply with our primary endpoint which was to establish the correlation between the prechoroidal cleft features and the disease activity and allowed us to include eyes that were already on treatment with anti-VEGF agents. Further prospective studies are necessary to confirm our results and investigate the possible use of the prechroidal cleft as a sign to guide re-treatment criteria in nAMD.

In conclusion, we found a significant increase of the prechoroidal cleft in association with MNV reactivation and a reduction of its size following treatment. Our results suggest that prechoroidal cleft could have a stronger correlation with MNV exudation compared to the PED and that it should be considered a sign of activity in nAMD. The appearance of a new prechoroidal cleft or an increase in its size in the context of a neovascular lesion could be considered an important biomarker to guide treatment decisions. Further studies are needed to investigate the role of prechoroidal cleft and its fluctuations in absence of other signs of disease activity.

## Supplementary Information

Below is the link to the electronic supplementary material.

Supplementary file1 (DOC 46 kb)

## References

[1] Miller JW (2013). Age-related macular degeneration revisited–piecing the puzzle: the LXIX Edward Jackson memorial lecture. Am J Ophthalmol.

[2] Lalwani GA, Rosenfeld PJ, Fung AE (2009). A variable-dosing regimen with intravitreal ranibizumab for neovascular age-related macular degeneration: year 2 of the PrONTO Study. Am J Ophthalmol.

[3] Silva R, Berta A, Larsen M (2018). Treat-and-extend versus monthly regimen in neovascular age-related macular degeneration: results with ranibizumab from the TREND study. Ophthalmology.

[4] Mrejen S, Sarraf D, Mukkamala SK, Freund KB (2013). Multimodal imaging of pigment epithelial detachment: a guide to evaluation. Retina.

[5] Spaide RF, Jaffe GJ, Sarraf D (2020). Consensus nomenclature for reporting neovascular age-related macular degeneration data. Ophthalmology.

[6] Mukai R, Sato T, Kishi S (2014). A hyporeflective space between hyperreflective materials in pigment epithelial detachment and Bruch’s membrane in neovascular age-related macular degeneration. BMC Ophthalmol.

[7] Spaide RF (2009). Enhanced depth imaging optical coherence tomography of retinal pigment epithelial detachment in age-related macular degeneration. Am J Ophthalmol.

[8] Nagiel A, Freund KB, Spaide RF (2013). Mechanism of retinal pigment epithelium tear formation following intravitreal anti-vascular endothelial growth factor therapy revealed by spectral-domain optical coherence tomography. Am J Ophthalmol.

[9] Rahimy E, Freund KB, Larsen M (2014). Multilayered pigment epithelial detachment in neovascular age-related macular degeneration. Retina.

[10] Singh SR, Lupidi M, Mishra SB (2020). Unique optical coherence tomographic features in age-related macular degeneration. Surv Ophthalmol.

[11] Kim JH, Chang YS, Kim JW (2018). Prechoroidal cleft in type 3 neovascularization: incidence, timing, and its association with visual outcome. J Ophthalmol.

[12] Kim JM, Kang SW, Son DY, Bae K (2017). Risk factors and clinical significance of prechoroidal cleft in neovascular age-related macular degeneration. Retina.

[13] Invernizzi A, Benatti E, Cozzi M (2018). Choroidal structural changes correlate with neovascular activity in neovascular age related macular degeneration. Invest Ophthalmol Vis Sci.

[14] Sadda SR, Guymer R, Holz FG (2018). Consensus definition for atrophy associated with age-related macular degeneration on OCT: classification of atrophy Report 3. Ophthalmology.

[15] Souied EH, Addou-Regnard M, Ohayon A (2020). Spectral-domain optical coherence tomography analysis of fibrotic lesions in neovascular age-related macular degeneration. Am J Ophthalmol.

[16] Querques G, Capuano V, Frascio P (2015). Wedge-shaped subretinal hyporeflectivity in geographic atrophy. Retina.

[17] Kumar JB, Stinnett S, Han JIL, Jaffe GJ (2020). Correlation of subretinal hyperreflective material morphology and visual acuity in neovascular age-related macular degeneratioN. Retina.

[18] Lai T-T, Hsieh Y-T, Yang C-M (2019). Biomarkers of optical coherence tomography in evaluating the treatment outcomes of neovascular age-related macular degeneration: a real-world study. Sci Rep.

[19] He L, Silva RA, Moshfeghi DM (2016). Aflibercept for the treatment of retinal pigment epithelial detachments. Retina.

[20] Busbee BG, Ho AC, Brown DM (2013). Twelve-month efficacy and safety of 0.5 mg or 2.0 mg ranibizumab in patients with subfoveal neovascular age-related macular degeneration. Ophthalmology.

[21] Dugel PU, Koh A, Ogura Y (2020). HAWK and HARRIER: phase 3, multicenter, randomized, double-masked trials of brolucizumab for neovascular age-related macular degeneration. Ophthalmology.

[22] Schmidt-Erfurth U, Waldstein SM, Deak G-G (2015). Pigment epithelial detachment followed by retinal cystoid degeneration leads to vision loss in treatment of neovascular age-related macular degeneration. Ophthalmology.

[23] Cho HJ, Kim KM, Kim HS (2016). Response of pigment epithelial detachment to anti-vascular endothelial growth factor treatment in age-related macular degeneration. Am J Ophthalmol.

[24] Keane PA, Patel PJ, Liakopoulos S (2012). Evaluation of age-related macular degeneration with optical coherence tomography. Surv Ophthalmol.

[25] Schmidt-Erfurth U, Waldstein SM (2016). A paradigm shift in imaging biomarkers in neovascular age-related macular degeneration. Prog Retin Eye Res.

[26] Khurana RN, Rahimy E, Joseph WA (2019). Extended (every 12 weeks or longer) dosing interval with intravitreal aflibercept and ranibizumab in neovascular age-related macular degeneration: post hoc analysis of VIEW trials. Am J Ophthalmol.

[27] Nguyen V, Puzo M, Sanchez-Monroy J, et al (2020) Association between anatomical and clinical outcomes of neovascular age-related macular degeneration treated with anti-VEGF. Retina Publish Ahead of Print: 10.1097/IAE.0000000000003061 10.1097/IAE.0000000000003061 PMC8210784 33332811

[28] Guymer RH, Markey CM, McAllister IL (2019). Tolerating subretinal fluid in neovascular age-related macular degeneration treated with ranibizumab using a treat-and-extend regimen: FLUID study 24-month results. Ophthalmology.

[29] Simader C, Ritter M, Bolz M (2014). Morphologic parameters relevant for visual outcome during anti-angiogenic therapy of neovascular age-related macular degeneration. Ophthalmology.

[30] Chakravarthy U, Havilio M, Syntosi A (2021). Impact of macular fluid volume fluctuations on visual acuity during anti-VEGF therapy in eyes with nAMD. Eye (Lond).

