# Supplementary material for: Prechoroidal cleft thickness correlates with disease activity in neovascular age-related macular degeneration
Source: Graefes Arch Clin Exp Ophthalmol. 2021 Sep 7;260(3):781–9. doi: 10.1007/s00417-021-05384-w (PMC8850287; doi:10.1007/s00417-021-05384-w)
Supplement: Supplementary file 1 — Supplementary file1 (DOC 46 kb) [file 417_2021_5384_MOESM1_ESM.doc]

**Supplementary Table 1.** Intraclass correlation coefficient measured between two independent readers for each anatomical outcome

| **SD-OCT Features** | **T0** | | **T1** | | **T2** | |
| --- | --- | --- | --- | --- | --- | --- |
|  | **ICC** | **95% CI** | **ICC** | **95% CI** | **ICC** | **95% CI** |
| Subfoveal prechoroidal cleft maximum height | 0.81 | 0.78 – 0.86 | 0.88 | 0.84 – 0.93 | 0.87 | 0.82 – 0.92 |
| Subfoveal prechoroidal cleft width | 0.85 | 0.79 – 0.89 | 0.91 | 0.87 – 0.96 | 0.90 | 0.85 – 0.94 |
| Greatest prechoroidal cleft height | 0.83 | 0.79 – 0.89 | 0.88 | 0.86 – 0.93 | 0.86 | 0.82 – 0.91 |
| Greatest prechoroidal cleft linear dimension | 0.89 | 0.86 – 0.94 | 0.85 | 0.80 – 0.88 | 0.82 | 0.79 – 0.86 |
| Subfoveal PED maximum height | 0.92 | 0.91 – 0.95 | 0.90 | 0.88 – 0.92 | 0.92 | 0.90 – 0.96 |
| Subfoveal PED width | 0.89 | 0.88 – 0.92 | 0.87 | 0.83 – 0.89 | 0.87 | 0.84 – 0.89 |
| Greatest PED height | 0.90 | 0.86 – 0.94 | 0.93 | 0.87 – 0.96 | 0.91 | 0.89 – 0.95 |
| Greatest PED linear dimension | 0.87 | 0.82 – 0.91 | 0.88 | 0.84 – 0.91 | 0.89 | 0.87 – 0.93 |
| SD-OCT: spectral domain optical coherence tomography; PED: pigment epithelial detachment; ICC: intraclass correlation coefficient; CI: confidence interval. | | | | | | |
